# Nonnegligible causes of symptoms of acute lower extremities——3 cases of May-Thurner syndrome with deep vein thrombosis

**DOI:** 10.1186/s12959-021-00278-7

**Published:** 2021-04-19

**Authors:** Yi Sun, Shenghan Song

**Affiliations:** Department of Vascular Surgery, Beijing Chaoyang Hospital, Capital Medical University, Beijing, 100020 China

**Keywords:** May-Thurner syndrome, Deep vein thrombosis, Stent

## Abstract

**Background:**

May-Thurner syndrome is a kind of disease caused by the compression of the left common iliac vein. It is one of the causes of incomplete venous valves and superficial varicose veins in lower limbs, and is also a potential factor of acute deep vein thrombosis (DVT).

**Method:**

Here 3 cases are diagnosed as May-Thurner syndrome at different ages.

**Case presentations:**

**1.** A 35-year-old female patient was hospitalized with swelling of the left lower limb for 1 week. Computed tomography (CT) showed compression of the left common iliac vein with thrombosis. May-Thurner syndrome was diagnosed and catheter-directed thrombolysis was performed. **2.** A 37-year-old male patient came to our hospital due to sudden swelling of the right lower extremity and pain for 3 days. Computed tomography showed compression of the left common iliac vein and deep venous thrombosis (DVT) of the right iliac vein. May-Thurner syndrome was diagnosed. The patient was performed with inferior vena cava (IVC) filter implantation, catheter-directed thrombolysis and balloon angioplasty for right iliac vein. And the patient recovered well; **3.** A 55-year-old female patient came to our hospital with swelling and discomfort in the left lower extremity for 3 days. Computed tomography showed stenosis of the left common iliac vein with deep vein thrombosis. May-Thurner syndrome was diagnosed, balloon dilation and stent implantation were performed. During 3 years of follow-up, there was no swelling or new thrombosis in her lower limbs.

**Conclusion:**

When encountering unexplained deep vein thrombosis, iliac vein compression syndrome should be considered and treated in time to prevent the recurrence of thrombosis. Catheter-directed thrombolysis can relieve symptoms and stenting placement is the optimal way to relieve stenosis, supplemented by long-term anticoagulation therapy and graduated compression stockings.

## Introduction

**Fig. 1:**
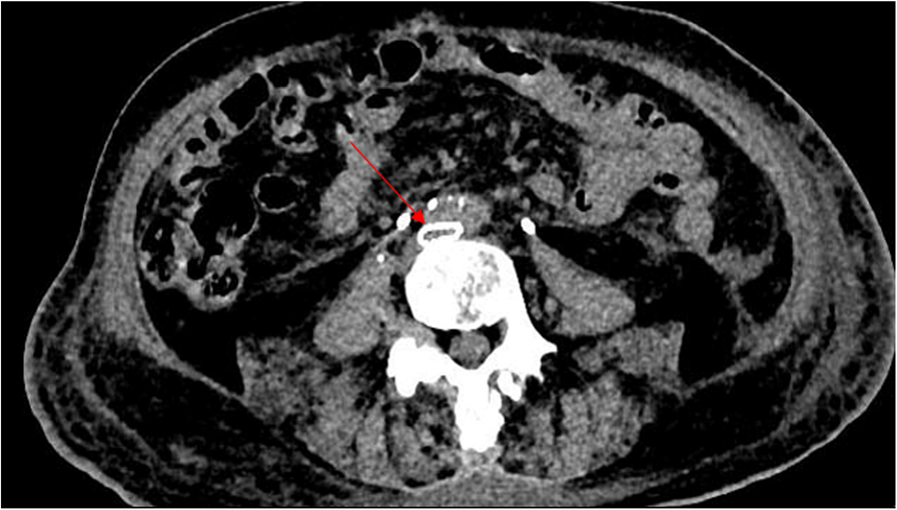
A patient was performed stent implantation in left iliac vein one year ago and recent CT result showed that the left iliac vein was compressed again

Iliac vein compression syndrome (IVCS, May-Thurner syndrome), also known as Cockett syndrome, is mostly caused by the right iliac artery or vertebral body compressing the left iliac vein (Fig. 1). It is one of the causes of venous valve insufficiency and varicose veins in the lower extremities and an important potential factor for secondary iliac-femoral vein thrombosis [1]**.** The main clinical symptoms are deep vein thrombosis, edema, pain, varicose veins and ulcers in the left lower limb [2]. Deep vein thrombosis (DVT) usually originates from the tip of the venous valve and can occur in any deep vein (more common on the left). It is common in patients with trauma, major surgery, such as total joint replacement, and stroke patients [3, 4]. The deep vein thrombosis caused by IVCS may be caused by the close contact of the right common iliac artery, lumbar spine, sacrum and left common iliac vein, and repeated stimulation of the vein wall by arterial pulsation, causing chronic damage to the vein and tissue reaction. Because the etiology is often overlooked, the treatment of deep vein thrombosis is imperfect, and it is easy to relapse [1, 2].

The prevalence of May-Thurner syndrome in the population is relatively high. Among them, women are more common. The probability of simultaneous compression of both lower limbs is 46%, but only 2 ~ 5% produce symptoms (acute iliac-femoral vein thrombosis, varicose veins, etc.), so it is often overlooked by clinicians especially for physicians [5]**.**

We report 3 patients of May-Thurner syndrome with deep vein thrombosis, summarizing their clinical symptoms, imaging findings, treatment process and follow-up, which may promote our understanding of May-Thurner syndrome, and play a certain guiding role in future treatment. IRB approval was not required, Ethics approval and consent to participate.

## Case presentations

### Case1

**Fig. 2:**
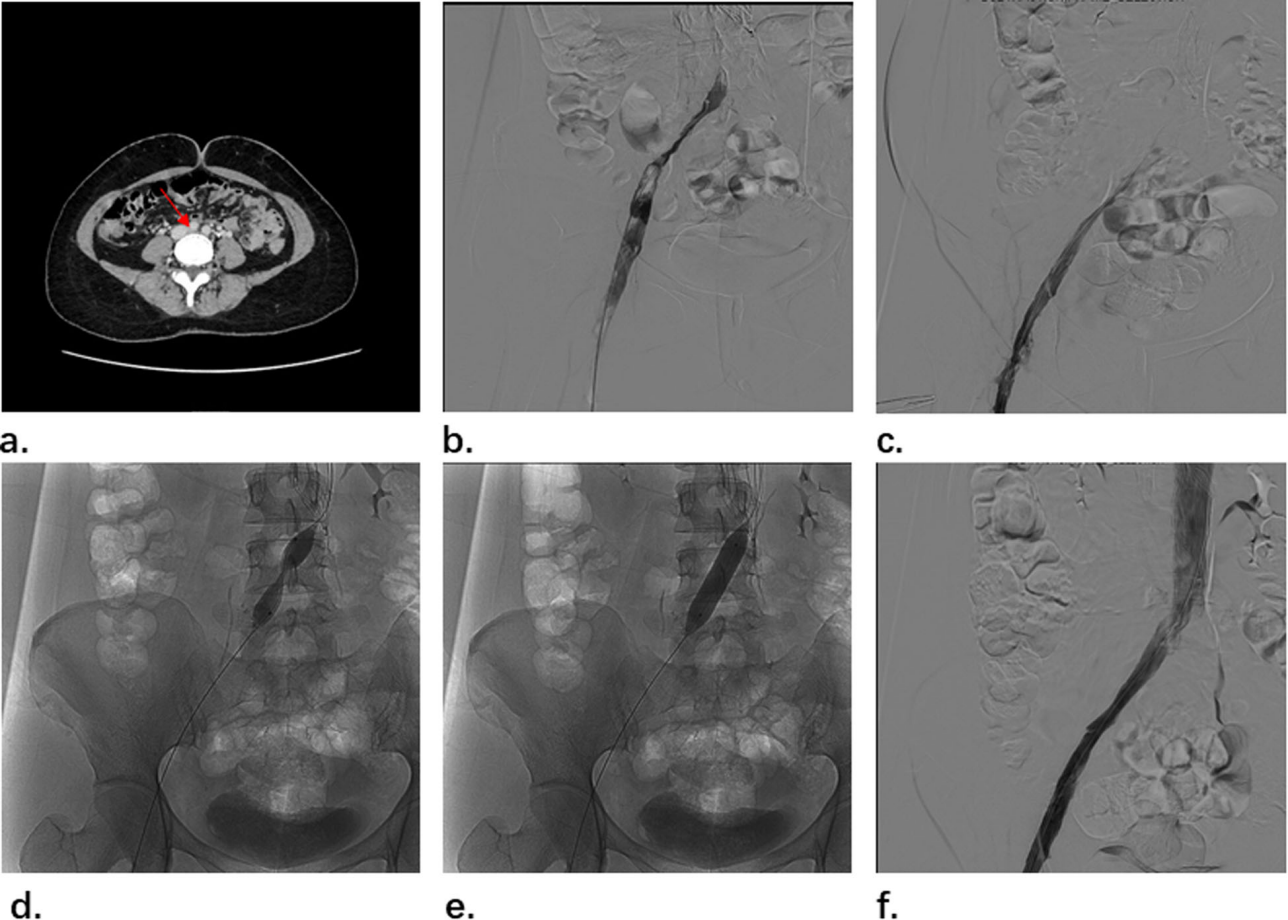
**a** CT showed May-Thurner syndrome. **b** venography showed that significant stenosis in left common iliac vein. **c** Three days later, the venography showed that the thrombus was almost completely dissolved. **d**-**e** 12 mm*40 mm PTA balloon dilatation of left common iliac vein. **f** left common iliac vein stenosis relieved and the inferior vena cava was visible

A 35-year-old female patient weighing 47 kg experienced swelling and pain in the left thigh 1 week ago with no obvious cause, and could not be relieved after rest. The ultrasound was performed at the local hospital 3 days ago, indicating the left iliac vein thrombosis. Symptoms had not improved after anticoagulation treatment. Then she was transferred to our hospital for further treatment and computed tomography (CT) showed May-Thurner syndrome with deep vein thrombosis of left lower extremities (Fig. 2a). The patient had no history of lower extremity disease, and she had no personal or family history of hypercoagulable disorder, but she had taken contraceptive pills for 3 years and D-dimer was 2.14 mg/L on admission.

The patient accepted enoxaparin sodium (4000 AXa IU every 12 h) for anticoagulant therapy in the perioperative period. Then the patient received a right femoral vein puncture in the supine position (the delay between the thrombotic event and the procedure was 11 days). Firstly, a filter was implanted in the inferior vena cava; then the patient was changed to the prone position and left popliteal vein puncture was performed for venography of left lower extremity. After thrombus aspiration, the significant narrow left common iliac vein was exposed (Fig. 2 b). Then urokinase (1 million u/day) was pumped for thrombolytic treatment after procedure. 3 days later, the result of venography showed that the thrombus was almost completely dissolved and the severe stenosis in left common iliac vein was exposed (Fig. 2 c). Then a 12 mm*40 mm PTA balloon was used to expand it (Fig. 2 d-e). Further venography showed the inferior vena cava was visible (Fig. 2 f). Considering that the thrombolytic efficacy was satisfactory and the patient was young, we did not perform stent implantation for her. 10 days later, the patient discharged and the D-dimer was 2.07 mg/L. The patient was given rivaroxaban for anticoagulant therapy (15 mg two times a day in the first three weeks, then 20 mg one time a day orally) and graduated compression stockings (30–40 mmHg) for at least one year after admission. 2 weeks later, we removed the filter from the patient.

#### Case2

**Fig. 3:**
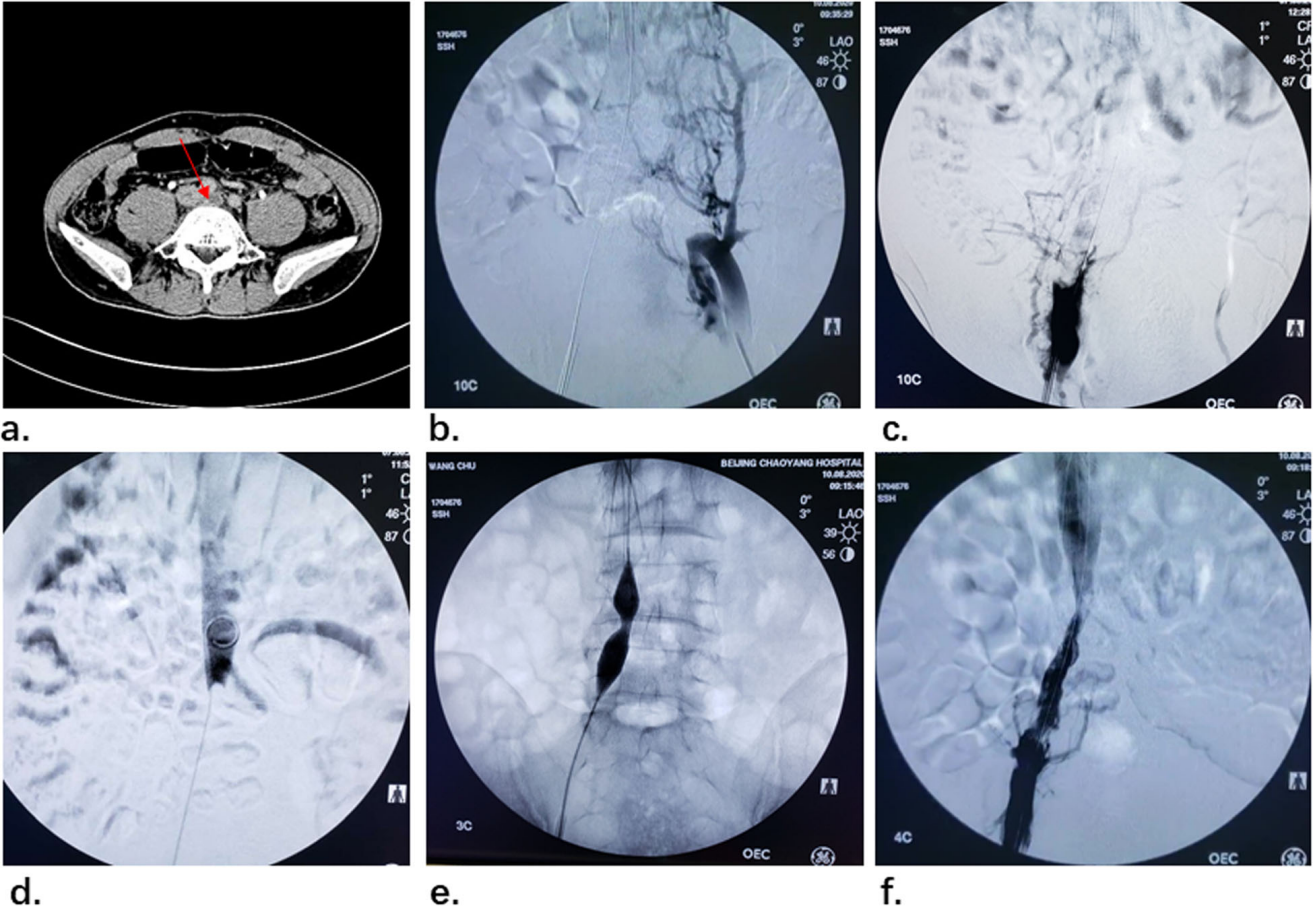
**a** CT shows May-Thurner syndrome. **b** the angiography showed complete occlusion of the left common iliac vein. **c**-**d** the middle and upper segments of right iliac vein were filled with a large amount of thrombus. **e** expanding right iliac vein with 14 mm*4 mm XXL balloon. (f) the stenosis of the right iliac vein and inferior vena cava was relieved

A 37-year-old male patient weighing 64 kg worked in front of a computer for more than 7 h a day. The patient experienced swelling and pain in his right lower limb 3 days ago, so he came to our hospital for treatment. The symptoms of left lower limb were not obvious. Computed tomography indicated thrombosis of the right iliac vein, thrombosis of the left iliac vein and inferior vena cava, and stenosis due to compression of the left iliac vein by right iliac artery. May-Thurner syndrome along with DVT was diagnosed. (Fig. 3 a) The patient had no history of trauma, surgery and lower extremity disease, and he had no personal or family history of hypercoagulable disorder, The D-dimer was 10.3 mg/L on admission.

The patient accepted enoxaparin sodium (6000 AXa IU every 12 h) for anticoagulant therapy in the perioperative period. The venography of the lower extremity showed complete occlusion of the left common iliac vein (Fig. 3 b). The lower end of the right iliac vein was visible and the middle and upper segments were filled with a large amount of thrombus (Fig. 3c-d). The time between the thrombotic event and the procedure was 9 days. Firstly, a filter was placed in the inferior vena cava. A thrombolytic catheter was placed and urokinase (1 million u/day) was pumped for thrombolytic therapy. Three days later, we used a 14 mm*4 mm XXL balloon to expand the stenosis segment of right iliac vein after thrombolytic therapy (Fig. 3 e), the patient complained of pain in right waist during the procedure. The balloon could not be fully opened. We considered it was caused by local old lesions. We insisted on expanding it for 2 min. The angiography showed that the stenosis of the right iliac vein and inferior vena cava was quite relieved (Fig. 3 f). In addition, complete occlusion of left common iliac vein was showed. Because the tissue of occluded segment was tough and the guide wire could not pass through, the further procedure was stopped to prevent possible blood vessel rupture and bleeding. At the time of discharge, the D-dimer had dropped to 1.77 mg/L. Treatment was the same as case 1 after admission. Outpatient examination after 6 weeks, the swelling of both lower limbs disappeared completely, and the D-dimer had dropped to 0.2 mg/L. Sixty-four days later, we removed the filter from the patient, and the D-dimer was 0.31 mg/L.

### Case 3

**Fig. 4:**
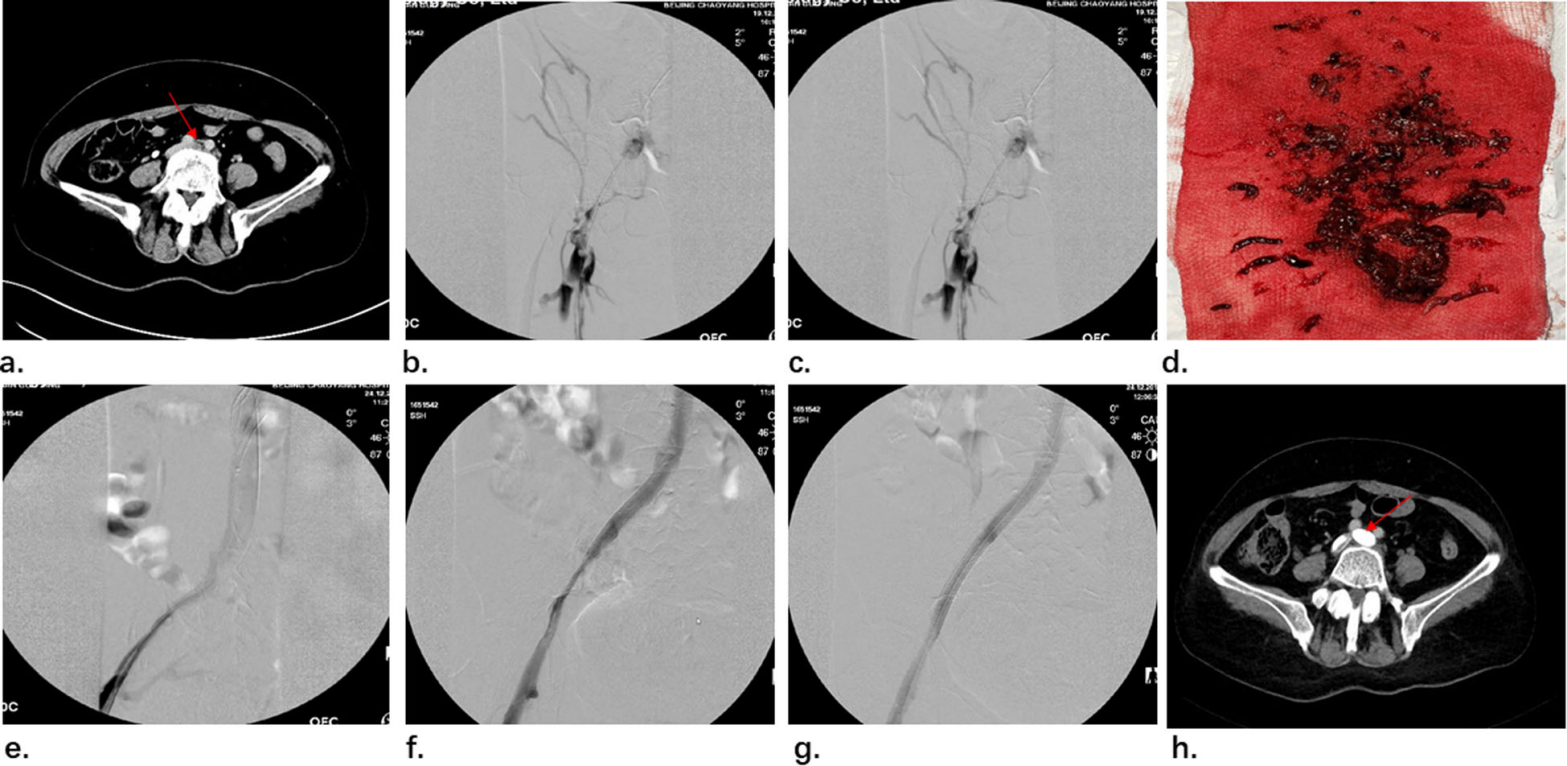
**a** CT showed May-Thurner syndrome. **b** the left iliac vein was full of thrombus. **c** compression and occlusion of the left common iliac vein were observed after thrombus aspiration (**d**) the blood clot that was aspirated out (**e**) compression of the left common iliac vein were showed after catheter thrombolysis and expansion by 12 mm*40 mm XXL balloon. **f** the first stent. **g** the second stent. **h** CT image of left iliac vein after 1 year

A 57-year-old female patient underwent surgical treatment for varicose veins 10 days ago at the local hospital. She experienced swelling and discomfort in her left lower extremity 3 days ago. She was not able to walk easily and could not be relieved after rest, then she was transferred to our hospital for further treatment. Computed tomography indicated deep venous thrombosis in the left lower extremity and significant stenosis of the left common iliac vein and compression by right iliac artery. May-Thurner syndrome with deep vein thrombosis was diagnosed (Fig. 4 a). The patient had a varicose vein in the left lower extremity for more than 10 years and had a great saphenous vein high ligation and exfoliation at the referring local hospital 10 days ago. and she had no personal or family history of hypercoagulable disorder, The D-dimer was 8.3 mg/L on admission.

The patient received enoxaparin sodium (6000 AXa IU every 12 h) for anticoagulant therapy immediately. We performed lower extremity angiography for the patient. The delay between the thrombotic event and the procedure was 9 days. A filter was placed in the inferior vena cava. Then urokinase (1 million u/day) was pumped. The venography after 2 days showed that the residual amount of thrombus decreased significantly. Three days later, venography showed that the stenosis in left common iliac vein was exposed clearly. Then a 12 mm*40 mm XXL balloon was used for expanding the stenosis two times, but the stenosis in left common iliac vein recurred due to elastic retraction (Fig. 4 e). So, stent implantation was decided and two 14 mm*60 mm and 12 mm*60 mm stents were placed in the left iliac vein (Fig. 4 f-g). The venography showed that the stenosis was completely relieved and the blood flow was unobstructed. The filter was taken out and the angiography showed that the inferior vena cava had smooth blood circulation. The CT showed that the stenosis of iliac vein was relieved (Fig. 4 h) and the D-dimer had dropped to 0.25 mg/L. Treatment was the same as case 1 after discharge. 2 weeks later, we removed the filter from the patient. No symptoms of lower extremities appeared during follow-up after 1 year and recent result of D-dimer was 0.32 mg/L.

## Discussion

In 1965, Cockett [6] first proposed and systematically explained the concept of iliac vein compression syndrome. It was also speculated that the patient would not have symptoms for a long time, which may be related to the appearance of collateral vein. The prevalence of May-Thurner syndrome in the population is relatively high. Among them, women are more common. The probability of simultaneous compression of both lower limbs is 46%, but only 2 ~ 5% produce symptoms (acute iliac-femoral vein thrombosis, varicose veins, etc.), so it is often overlooked by clinicians (especially for physicians). When abdominal or chest pressure increases, prolonged bed rest, standing or sitting, the risk of DVT will increase in the cases with MTS, but the incidence of pulmonary embolism is low because the compressed left iliac vein forms a barrier [7, 8].

Currently, there is no authoritative guideline to guide the diagnosis and treatment of May-Thurner syndrome. Most patients are admitted to the hospital for deep vein thrombosis, and Doppler vascular ultrasonography (DVUS) is a simple, convenient and low-cost examination method for diagnosing DVT of lower extremities, and it can also accurately evaluate the degree of disease, and show the anatomical relationship between iliac arteries and iliac veins. It also has a certain guiding effect on the placement of intravenous stents [9, 10]. Digital subtraction angiography (DSA) has a unique effect on the visualization of the iliac vein, but it cannot clearly show the relationship between the iliac vein and the iliac artery, and it is of little significance in the diagnosis of iliac vein compression. Computed tomography venography and magnetic resonance venography (MRV) can clearly show the relationship between the iliac vein and the iliac artery. It has unique advantages for the diagnosis of May-Thurner syndrome, and CT also helps to rule out obstruction of the left iliac vein or inferior vena cava caused by pelvic mass or lymph node disease [11–14]. However, because this is an invasive examination that requires the use of a large amount of contrast medium and has a certain degree of radiation, it is not suitable for pregnant women, and it is also dangerous to patients with renal impairment.

At present, endovascular treatment is the optimal treatment method for May-Thurner syndrome, including catheter thrombolysis, mechanical thrombus aspiration, IVC filter, balloon dilatation, stent implantation [15, 16]. Among which, catheter thrombolysis and mechanical thrombus aspiration are used for lower extremity venous thrombosis caused by iliac vein compression. After thrombolysis, the effect can be observed through venography. IVC filter can reduce the incidence of pulmonary embolism. The combination of balloon expansion and stent implantation is an effective treatment for iliac vein stenosis. Compared with traditional therapy (surgery), it has the advantages of minimal invasiveness, low risk, and low recurrence rate. Regarding the indications for stent implantation, Sang [17] believed that stent should be implanted when the degree of stenosis exceeds 70%, and when the stenosis is between 50 and 70%, It depends on the patients’ condition. A study by Jae Young Park [18] showed that in 55 patients after stent implantation, the patency rate of iliac vein stent implantation was 95.8% at 6 months, 87.5% at 12 months, and 84.3% at 24 months. Four patients had recurred thrombotic occlusion during follow-up (7.8%). Hager [19] found that the patency rate of 36 months after stent implantation was at least 91%. Robert R [20] also found that the stent diameter has a significant impact on the patency rate of the iliac vein stent, stent diameter is proportional to patency rate. Even with stent placement, postoperative anticoagulation therapy is necessary, which can not only effectively alleviate the prognosis, but also prevent relapse [7]**.** Timely treatment of iliac vein stenosis can effectively prevent the recurrence of DVT in the lower limbs, thereby preventing the occurrence of post-thrombotic syndrome (PTS) [21]**.**

Case 1 is a female patient with acute symptoms. After thrombolytic treatment, her symptoms of lower extremity disappeared, so stent implantation of left iliac vein was not performed; Case 2 is a young man with typical symptoms in the right lower extremity. After medication and thrombolytic treatment, his symptoms quickly relieved. The stenosis of right iliac vein was considered to be caused by old thrombosis. The patient was young and refused further stent implantation. The symptoms of his lower extremity disappeared completely when he was discharged from the hospital. Compared with Case 1, it was more difficult for Case 2 to dissolve the thrombus of lower extremity, which may be related to old thrombosis. Thrombolytic therapy was more suitable for patients with acute thrombosis. It can dissolve fresh thrombus, expose old lesions, and allow further endovascular treatment such as balloon dilation or stent implantation.

Case 3 had a history of severe varicose veins in lower extremities with pigmentation for 10 years, and DVT occurred shortly after varicose great saphenous vein surgery. The presence of May-Thurner syndrome was detected by CT and venography. We speculated that the etiology of varicose saphenous vein in this patient might be related to May-Thurner syndrome. The risk of DVT cannot be ignored if such patients undergo surgery or do not take anticoagulation and other preventive measures after surgery. Therefore, supplementary examinations should be considered for severe lower extremity varicose veins of patients with limb swelling and pigmentation. For case 3, May-Thurner syndrome can also cause lower extremity varicose veins**,** the surgical injury may not only induce acute deep vein thrombosis [22, 23]. but also may cause varicose vein recurrence due to neglect of iliac vein compression syndrome. Treatment of May-Thurner syndrome, including balloon dilatation of the left iliac vein or stent implantation, followed by treatment of lower extremity varicose veins may be a more appropriate procedure. We performed mechanical aspiration of thrombus, thrombolysis, balloon dilatation of the left iliac vein, and stent implantation (stenosis recurrences due to elastic retraction of the local tissue after balloon dilatation). There was no recurrence of DVT during 1-year follow-up. Varicose of the great saphenous vein due to May-Thurner syndrome is not common. Clinicians often mistakenly think it is simple varicose veins, thus neglecting corresponding etiological examinations and anticoagulant measures during perioperative period. This may result in acute deep vein thrombosis or recurrence of varicose veins. Some scholars [24] find that the prior endovascular treatment can also reduce the recurrence rate of varicose veins.

The characteristics of May-Thurner syndrome in our patients can be summarized as follows: 1) Symptoms of May-Thurner syndrome are usually atypical and often neglected by clinical physicians. We should pay attention to it to avoid unexpected complications; 2) Thrombolytic therapy was more suitable for patients with acute thrombosis; 3) If the stenosis is mild, simple thrombolytic therapy and balloon dilatation should be enough. If the stenosis is severe, stent implantation should be considered to relieve the stenosis and reduce the recurrence rate of thrombosis.

## Conclusion

Symptoms of May-Thurner syndrome are not usually obvious, so it is often ignored by clinicians. When patients have unexplained lower limb swelling and deep vein thrombosis, we should consider the possibility of May-Thurner syndrome and perform diagnosis and treatment. We should pay more attention to it to avoid unexpected acute deep vein thrombosis.

## Data Availability

The datasets obtained and analyzed in the current study are available from the corresponding author on reasonable request.

## Abbreviations

DVT: Deep vein thrombosis
CT: Computed tomography
IVC: Inferior vena cava
IVCS: Iliac vein compression syndrome
DVUS: Doppler vascular ultrasonography
DSA: Digital subtraction angiography
MRV: Magnetic resonance venography
PTS: Post-thrombotic syndrome
